# Validation of Ayurvedic formulations in animal models requires stringent scientific rigor

**DOI:** 10.4103/0975-9476.72607

**Published:** 2010

**Authors:** S. C. Lakhotia

**Affiliations:** *Department of Zoology, Cytogenetics Laboratory, Banaras Hindu University, Varanasi - 221 005, India. E-mail: lakhotia@bhu.ac.in*

Sir,

The paper by Priyadarshini, *et al*. entitled “Increase in *Drosophila melanogaster* longevity due to rasayana diet: Preliminary results,” published in April 2010 issue of *Journal of Ayurveda and Integrative Medicine* concludes that dietary supplement of a specially formulated “rasayana” to *Drosophila melanogaster* flies significantly enhances their lifespan and that this observation seemingly agrees with what is stated about rasayana in Ayurvedic literature. This can be an important finding. Unfortunately, however, the present paper falls significantly short of a scientific analysis and reporting.

Although strong claims are made in the rather verbose Introduction” and “Discussion” sections, the real description of the methods is very limited. The major limitations are as follows:

- Neither the nature of the rasayana nor its method of preparation is described in the paper. Authors use terms like “*Drosophila rasayana*” or “*insect rasayana,*” which certainly do not exist in Ayurvedic texts! For a scientific study, one would like to know how it differs from or resembles the typical rasayana preparations.
- How much rasayana was actually available to the flies and how was it mixed with food? With respect to the diet, authors state “Controls received 1 drop (10 μl) of bacteriological grade yeast solution as food; experimental cultures received 1 drop *Drosophila* rasayana food supplement. No dietary restrictions were imposed. Flies could feed *ad libidum*.” This does not provide any information about the concentration or the nature of rasayana given. Nor is any mention made of the concentration of yeast solution nor the quantity of food to which the “1 drop” of yeast solution or the rasayana supplement was added. What quantity of the standard fly food was provided in each vial?

With such limited information about the methods of study, no one can reproduce the results for verification! A basic requirement of a scientific analysis, whether “preliminary” or more definitive, is that the materials and method used in the study should be explicit enough so that those interested can use the same for verification or for more advanced studies. In the present case, this is not possible because such essential information has not been provided. It is surprising that on the basis of “a preliminary study,” as title of the paper states, authors claim their “finding” that the rasayana-fed flies displayed significantly increased longevity is as definitive “as a law of physics or chemistry”! It is likely that the life span is indeed enhanced by the “rasayana” feeding. However, establishing it, especially from the viewpoint of implications for human society, requires more in depth data and analysis together with all the relevant details. Enough data must be provided to establish that the modified *rasayana* used by authors in the present study is essentially similar in its actions and composition to the traditional *rasayana*. Hiding the essential details is not justifiable in a scientific study.

In addition to the above serious limitations of experimental part of the study, there are factual errors relating to some scientific facts. For example, the mammalian *Xist* and *Drosophila Sxl* genes are not at all analogous since unlike the protein coding *Sxl gene* of *Drosophila*, the *Xist* gene is a noncoding one and is not involved in sex determination! Although both have roles in dosage compensation, their mechanisms of action are entirely different. Thus they are not even functional analogues. It is also not true to say that for every *Drosophila* gene, there are about four homologues in vertebrates. The *methuselah* mutant of *Drosophila* is a single-gene mutation rather than it being the result of selection of all genes involved in ageing in flies as seems to be implied in the statement of authors on p. 115. Such erroneous claims in a scientific paper can be very misleading indeed. Another seemingly trivial but important point is that the *P* value cannot be less than “zero” (or <0.000 as given in Table 2).

In recent years, there has been a welcome flurry of research activity to justify the principles and practice of Ayurveda and other traditional medical systems. There is no question that *Drosophila* is a very good model for studies directed to understand molecular basis of the Ayurvedic formulations. However, unless such studies are rationally planned and conducted with the required experimental rigor, the apparently “positive” results may actually be more damaging to the system which the authors would want to strengthen. The published study by Priyadarshini *et al*. under reference does not seem to meet the basic rigor of a scientific study. Publication of such poorly executed study, which is presented more in “popular” article style rather than a professional scientific report, is also not good for the journal.

